# Correlation Analysis of Gensini Score in Diabetic Patients with Coronary Heart Disease

**DOI:** 10.31083/j.rcm2411319

**Published:** 2023-11-17

**Authors:** Jinyue Qi, Yunzhe Wang, Zhiyu Liu, Fengyi Yu, Junnan Tang, Jinying Zhang

**Affiliations:** ^1^Department of Cardiology, The First Affiliated Hospital of Zhengzhou University, 450052 Zhengzhou, Henan, China; ^2^Henan Province Key Laboratory of Cardiac Injury and Repair, 450052 Zhengzhou, Henan, China; ^3^Henan Province Clinical Research Center for Cardiovascular Diseases, 450018 Zhengzhou, Henan, China; ^4^Clinical Big Data Center, The First Affiliated Hospital of Zhengzhou University, 450052 Zhengzhou, Henan, China

**Keywords:** diabetes mellitus, cardiovascular heart disease, Gensini score, MACCEs

## Abstract

**Background:**

Assessment of risk factors is essential for clinical diagnosis and prevention in patients with both diabetes mellitus (DM) and coronary heart disease (CHD). In the present study we investigated correlation of the Gensini score with the incidence of major adverse cardiac and cerebrovascular events (MACCEs) in patients with DM and CHD.

**Methods:**

A total of 802 DM patients with CHD admitted to the First Affiliated Hospital of Zhengzhou University and who met the inclusion criteria were enrolled in the study. The median follow-up time for these patients was 3000 days (range 382.5–3000). Receiver operating characteristic (ROC) curves for the Gensini score were generated and the area under the curve (AUC) was calculated. Patients were divided into two groups based on the Gensini score cut-off value. Univariate and multivariate Cox proportional hazard regression analysis was used to identify the risk factors associated with MACCEs. The incidence of MACCEs in the two groups was compared using Kaplan-Meier analysis.

**Results:**

The AUC of the ROC curve was 0.675. The maximum Youden’s index was 0.248 at a Gensini score cut-off value of 74.8605. This gave a sensitivity and specificity for the prediction of MACCE of 68.8% and 56%, respectively. A high Gensini score was a risk factor for MACCEs, and the incidence of MACCEs was significantly greater in the high Gensini score group compared to the low Gensini score group.

**Conclusions:**

A high Gensini score is a risk factor for patients with DM and CHD and is associated with a high incidence of MACCEs.

**Clinical Trial Registration:**

The details of study design are registered on http://www.chictr.org.cn (identifier: ChiCTR-2200055450).

## 1. Introduction 

Diabetes mellitus (DM) is a common disease worldwide, with increasing rates of morbidity and mortality. The number of adults with DM is projected to increase from 194 million in 2003 to nearly 380 million in 2025 [1]. The broadest diagnostic consensus of metabolic syndrome (MetSyn) is the presence of at least three of the five risk factors, namely hyperglycemia, hypertension, elevated triglyceride levels, low high-density lipoprotein cholesterol levels, and central obesity [2]. In a state of stress, MetSyn is prone to develop into clinical diabetes. DM is known to be a complex multifactorial disease. However, the risk of poor clinical outcome is greater in patients with clusters of multiple risk factors than in patients with only one risk factor. Moreover, the effects are not simply additive, but synergistically exacerbated. Thus, MetSyn increases the risk of coronary heart disease (CHD) and other cardiovascular diseases in DM patients [3, 4]. The risk of cardiovascular disease is increased 2–4-fold in diabetic individuals [5]. A great deal of previous research has shown that CHD is the major cause of mortality in DM patients [6]. It is now well-established that three quarters of diabetic patients aged >40 years will die from cardiovascular disease [7, 8]. Therefore, risk assessment and individualized treatment is essential for patients with early-stage DM combined with CHD [8, 9].

Atherosclerosis is a chronic inflammatory disease of the arteries and is the main cause of CHD, cerebral infarction, and other cardiovascular and cerebrovascular diseases that result in major adverse cardiac and cerebrovascular events (MACCEs) [10]. If the atherosclerotic plaque becomes unstable, ruptured or corroded, this can threaten the patients’ life. Many studies have established that DM is an independent risk factor for atherosclerosis. One study suggested that DM patients are more likely to develop atherosclerosis than individuals without DM [11]. A cross-sectional, observational, multi-center study showed that people with DM are more likely to develop plaque calcification and arteriosclerosis than those without DM due to higher blood viscosity and a high plaque burden [12]. Clinically, the Gensini score is a quantitative evaluation of the degree of coronary-artery stenosis, with the total score being positively correlated with the severity of the patient’s condition [13]. However, few studies have investigated the prognostic value of the Gensini score in patients with both DM and CHD. In the present study, we therefore evaluated the correlation between the Gensini score and clinical outcomes in DM patients with CHD.

## 2. Materials and Methods

### 2.1 Patients

A total of 831 patients with both DM and CHD and who were admitted to the Zhengzhou University’s First Affiliated Hospital between January 1, 2013 and January 31, 2018, were selected. Inclusion criteria were: (1) DM patients diagnosed with CHD; and (2) the availability of coronary angiography findings. Exclusion criteria were: (1) severe infections, autoimmune diseases, malignancies, primary kidney disease; and (2) incomplete data. Follow-up information was obtained via phone or revisit, with 29 patients being lost to follow-up. MACCEs were assessed during follow-up and included recurrent myocardial infarction, cardiogenic death, cardiac insufficiency, chest pain readmission, stroke, and all-cause death.

DM diagnosis criteria [5] were: fasting blood glucose ≥7.0 mmol/L; 2 h postprandial blood glucose ≥11.1 mmol/L; or previously diagnosed DM. The diagnostic threshold for CHD was computed tomography angiography or digital subtraction angiography revealing coronary stenosis >50%. The Gensini score was used to evaluate the degree of coronary artery lesions according to the results of coronary angiography. The score was given according to the degree of vascular stenosis [13]: 0 points, no lesion found; 1 point, stenosis ≤25%; 2 points, stenosis of 25–50%; 4 points, stenosis of 50–75%; 8 points, stenosis of 75–90%; 16 points, stenosis of 90–99%; and 32 points for stenosis of 100%. Scores were then weighted according to the location of the vascular lesion: left main artery lesion, ×5; proximal anterior descending branch and proximal circumflex branch, ×2.5; middle anterior descending branch, ×1.5; right coronary artery, distal anterior descending branch, diagonal branch, posterior branch of left ventricle, blunt margin branch, ×1.0; other vascular lesions, ×0.5. The total score is the sum of the scores for each branch.

### 2.2 Conventional Clinical and Laboratory Indicators

The clinical data for 802 diabetic patients with CHD was recorded and analyzed, including gender, age, history of smoking, medication, hypertension, heart failure, and cerebrovascular disease. Venous blood (5 mL) was drawn for testing after fasting for at least 8 h. Blood tests included white blood cell (WBC) count, C-reactive protein (CRP), platelet values, neutrophil count, lymphocyte count, hemoglobin, glycosylated hemoglobin A1c (HbA1c), N-terminal pro-brain natriuretic peptide (NT-proBNP), creatinine, high-density lipoprotein (HDL), low-density lipoprotein (LDL), total cholesterol, triglycerides and Gensini score.

### 2.3 Statistical Analysis

The Gensini score was calculated using an online calculator. Statistical analysis was performed using IBM SPSS (v 24.0, IBM Corp., Chicago, IL, USA) software. Continuous data was expressed as the mean ± standard deviation (SD), while the Student’s *t*-test was used for comparison between groups. Categorical variables were compared with the Chi-square (χ^2^) test and were displayed as percentages. Multivariate Cox regression analysis was used to analyze the risk of MACCEs. The Kaplan-Meier method was used for survival analysis, and the log-rank test was run on the resulting curves. A *p* value of <0.05 was considered to represent statistical significance.

## 3. Results

### 3.1 Baseline Characteristics and Laboratory Results of Patients Groups with Low and High Gensini Scores

**Fig. 1.**
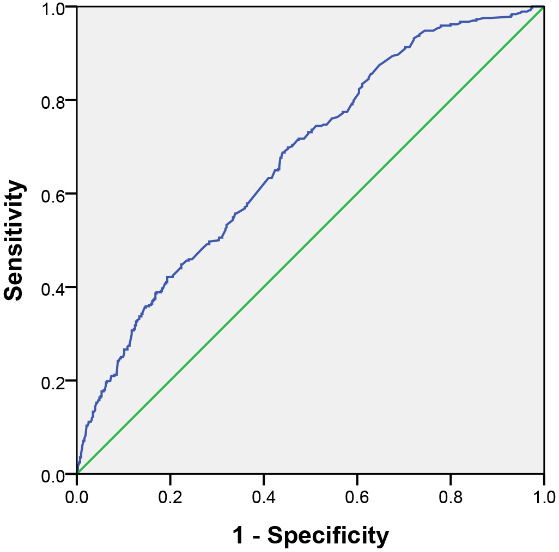
**ROC curve for the Gensini score**. ROC, receiver operating characteristic.

After excluding 29 patients who were lost to follow-up, data from 802 patients was available for analysis. The median follow-up time was 3000 days (382.5–3000). As shown in Fig. 1, the receiver operating characteristic (ROC) curve was used to calculate the sensitivity (Sen) and specificity (Spe) of the Gensini score for the prediction of MACCEs. Area under curve (AUC) is defined as the area under the ROC curve, with a value in the range of 0 to 1. The higher the AUC value, the better the classification effect of the model and the more accurate the detection of disease. As shown in Fig. 1, the AUC was calculated as 0.675 (95% CI: 0.638–0.711; *p* < 0.001), suggesting the classification effect of the Gensini score was good. The Sen, Spe, and Youden’s index (YI) were used to determine the ability of different scores to estimate the risk of MACCEs. The formula, *YI *= *Sen* + *Spe* – 1, was used to calculate the YI. The optimal trade-off between Sen and Spe was found when the YI was maximal. This occurred at a cut-off value of 74.8605. With this cut-off value, the Sen, Spe, and YI for the prediction of MACCE were 68.8%, 56% and 0.248, respectively. Thus, the incidence of MACCE was higher in patients with a score above this cut-off value.

Patients were classified into two groups according to the cut-off value: low Gensini score (≤74.8605) and high Gensini score (>74.8605). A total of 358 patients were in the low Gensini score group and 444 in the high Gensini score group. Age (*p* = 0.006), history of oral anti-diabetic drug use (*p* < 0.001), HbA1c level (*p* = 0.021), platelet count (*p* = 0.038) and creatinine level (*p* = 0.007) were significantly different between these two groups. Patients in the high Gensini score group had higher levels of HbA1c, platelet, Gensini score and anti-diabetic drug use, while patients in the low Gensini score group were older and had higher creatinine levels. Pertinent baseline characteristics and clinical laboratory results for the two groups are shown in Tables 1,2.

**Table 1.** **Baseline characteristics of patients with low or high Gensini scores**.

Variable		Low Gensini score group	High Gensini score group	*p*
		*n* = 358	*n* = 444	
Male/Female		265 (74.0%)/93 (26.0%)	330 (74.3%)/114 (25.7%)	0.923
Age/years (mean ± SD)		59.66 ± 11.88	57.40 ± 11.33	0.006**
	Age >60 years/Age ≤60 years	192 (53.6%)/166 (46.4%)	194 (43.7%)/250 (56.3%)	0.005**
Previous				
	Smoking	150 (41.9%)	197 (44.4%)	0.483
	Hypertension	212 (59.2%)	253 (57.0%)	0.524
	Heart failure	201 (56.1%)	265 (59.7%)	0.313
	Cerebrovascular disease	50 (14.0%)	63 (14.2%)	0.928
	Aspirin	292 (81.6%)	374 (84.2%)	0.317
	Beta-blockers	213 (59.5%)	287 (64.6%)	0.135
	Statins	305 (85.2%)	389 (87.6%)	0.319
	Oral anti-diabetic drug	122 (34.1%)	216 (48.6%)	<0.001***
	Insulin	107 (29.9%)	160 (36.0%)	0.066
	SGLT2	17 (4.7%)	30 (6.8%)	0.229
	Anti-hypertensive drug	197 (55.0%)	268 (60.4%)	0.181
	Calcium blockers	23 (6.4%)	39 (8.8%)	0.214

SGLT2, sodium-dependent glucose transporters 2; Low Gensini score group: ≤74.8605, High Gensini score group: >74.8605; **p < 0.01, *** *p* < 0.001.

**Table 2.** **Laboratory results for patients with low or high Gensini scores**.

Variable	Low Gensini score group	High Gensini score group	*p*
	*n = *358	*n* = 444	
WBC (${\mathrm{10}}^{9}$·$L^{–\,1}$)	9.40 ± 3.64	9.28 ± 3.95	0.671
Hemoglobin (g·$L^{–\,1}$)	132.19 ± 22.88	135.17 ± 21.72	0.072
HbA1c (%)	7.30 ± 1.74	7.61 ± 1.80	0.021*
Platelet (${\mathrm{10}}^{9}$·$L^{–\,1}$)	211.98 ± 64.73	222.01 ± 65.70	0.038*
Neutrophil (${\mathrm{10}}^{9}$·$L^{–\,1}$)	7.30 ± 4.72	6.94 ± 3.89	0.250
Lymphocyte (${\mathrm{10}}^{9}$·$L^{–\,1}$)	1.65 ± 1.61	2.10 ± 9.40	0.390
Triglyceride (mmol·$L^{–\,1}$)	1.75 ± 1.21	1.81 ± 1.19	0.431
Total cholesterol (mmol·$L^{–\,1}$)	4.04 ± 1.08	3.94 ± 1.05	0.192
Creatinine (µmol·$L^{–\,1}$)	87.58 ± 84.80	75.10 ± 43.86	0.007**
NT-proBNP (pg/${\mathrm{mL}}^{–\,1}$)	1856.44 ± 4421.33	1844.39 ± 3343.12	0.965
HDL (mmol·$L^{–\,1}$)	1.05 ± 0.31	1.02 ± 0.26	0.131
LDL (mmol·$L^{–\,1}$)	2.54 ± 0.95	2.47 ± 0.90	0.314
CRP (mg·$L^{–\,1}$)	22.07 ± 51.58	21.12 ± 51.79	0.819
Gensini score	49.06 ± 16.55	98.57 ± 22.59	<0.001***

WBC, white blood cell; HbA1c, glycosylated hemoglobin A1c; NT-proBNP, N-terminal pro-brain natriuretic peptide; HDL, high-density lipoprotein; LDL, low-density lipoprotein; CRP, C reactive protein; Low Gensini score: ≤74.8605, High Gensini score: >74.8605; *p < 0.05, **p < 0.01, ***p < 0.001.

### 3.2 Major Adverse Cardiac and Cerebrovascular Events in Patients with Low or High Gensini Scores

The endpoint for this study was MACCEs. These occurred in 115 (32.1%) of the low Gensini score patients and in 253 (57.0%) of the high Gensini score patients (*p* < 0.001). As shown in Table 3, recurrent myocardial infarction (*p* = 0.001), cardiogenic death (*p* = 0.003), cardiac insufficiency (*p* < 0.001), chest pain readmission (*p* < 0.001) and all-cause death (*p* = 0.004) of MACCEs were significantly different between the low and high Gensini score groups.

**Table 3.** **Gensini score correlates with the incidence of MACCEs**.

Variable		Low Gensini score	High Gensini score	*p*
		*n* = 358	*n* = 444	
MACCEs		115 (32.1%)	253 (57.0%)	<0.001***
	Recurrent myocardial infarction	32 (8.9%)	75 (16.9%)	0.001**
	Cardiogenic death	18 (5.0%)	47 (10.6%)	0.004**
	Cardiac insufficiency	22 (6.1%)	73 (16.4%)	<0.001***
	Chest pain readmission	82 (22.9%)	163 (36.7%)	<0.001***
	Stroke	2 (0.6%)	7 (1.9%)	0.142
	All-cause death	29 (8.1%)	66 (14.9%)	0.003**

Low Gensini score: ≤74.8605, High Gensini score: >74.8605; MACCEs, major adverse cardiac and cerebrovascular events; **p < 0.01, ***p < 0.001.

### 3.3 Univariate and Multivariate Cox Analysis

Baseline characteristics and laboratory results were evaluated using univariate models, as shown in Table 4. Patients with MACCEs were associated with increased odds of heart failure (HR [hazard ratio] 1.270, 95% CI: 1.029–1.567), insulin use (HR 1.263, 95% CI: 1.023–1.559), sodium-dependent glucose transporters 2 (SGLT2) use (HR 1.799, 95% CI: 1.258–2.574), HbA1c (HR 1.069, 95% CI: 1.011–1.131), lymphocytes (HR 1.012, 95% CI: 1.002–1.022), Gensini score (HR 1.014, 95% CI: 1.011–1.017), high Gensini score (Gensini score >74.8605) (HR 2.171, 95% CI: 1.741–2.707) and NT-proBNP (HR 1.000, 95% CI: 1.000–1.000). In contrast, MACCEs were associated with decreased odds of aspirin use (HR 0.628, 95% CI: 0.490–0.806) and of statin use (HR 0.545, 95% CI: 0.417–0.711).

The Gensini score and the high Gensini score (>74.8605) showed collinearity. These two factors were entered sequentially into multivariate Cox regression analysis along with other variables that showed significance (*p* < 0.05) in univariate Cox models. The results of multivariate Cox analysis showed that Gensini score, high Gensini score (>74.8605), history of SGLT2 and statin use, lymphocyte count, and the NT-proBNP level were risk factors for MACCEs in DM patients with CHD (Tables 5,6). The results showed increased odds for the Gensini score (HR = 1.013, 95% CI: 1.010–1.016, *p* < 0.001) and for high Gensini score (HR 2.270, 95% CI: 1.773–2.907, *p* < 0.001), thereby indicating their predictive value of adverse events.

**Table 4.** **Univariate Cox analysis results**.

Variable		Β	SE	Wald χ^2^	*p*	HR (95% CI)
Gender		–0.033	0.119	0.077	0.782	0.968 (0.767–1.221)
Age		0.129	0.104	1.538	0.215	1.138 (0.928–1.396)
Previous						
	Smoking	0.120	0.105	1.308	0.253	1.127 (0.918–1.384)
	Hypertension	–0.016	0.106	0.024	0.877	0.984 (0.800–1.210)
	Heart failure	0.239	0.107	4.940	0.026*	1.270 (1.029–1.567)
	Cerebrovascular disease	0.171	0.143	1.422	0.233	1.186 (0.896–1.570)
	Aspirin	–0.456	0.127	13.397	<0.001***	0.628 (0.490–0.806)
	Beta-blockers	–0.197	0.106	3.458	0.063	0.821 (0.667–1.011)
	Statins	–0.607	0.136	19.958	<0.001***	0.545 (0.417–0.711)
	Oral anti-diabetic drug	0.108	0.105	1.065	0.302	1.114 (0.907–1.368)
	Insulin	0.233	0.108	4.710	0.030*	1.263 (1.023–1.559)
	SGLT2	0.588	0.183	10.344	0.001***	1.799 (1.258–2.574)
	Anti-hypertensive drug	–0.076	0.098	0.600	0.438	0.927 (0.765–1.123)
	Calcium blockers	–0.334	0.215	2.402	0.121	0.716 (0.470–1.092)
WBC		0.010	0.015	0.492	0.483	1.011 (0.981–1.040)
Hemoglobin		–0.004	0.002	2.349	0.125	0.996 (0.992–1.001)
HbA1c		0.067	0.029	5.508	0.019*	1.069 (1.011–1.131)
Platelet		–0.002	0.001	3.192	0.074	0.998 (0.997–1.000)
Neutrophil		0.013	0.013	1.030	0.310	1.013 (0.988–1.040)
Lymphocyte		0.012	0.005	5.850	0.016*	1.012 (1.002–1.022)
Triglyceride		–0.082	0.048	2.860	0.091	0.922 (0.839–1.013)
Total cholesterol		–0.081	0.052	2.423	0.120	0.922 (0.832–1.021)
Creatinine		<0.001	0.001	0.002	0.965	1.000 (0.999–1.001)
NT-proBNP		<0.001	<0.001	9.814	0.002**	1.000 (1.000–1.000)
HDL		–0.127	0.191	0.442	0.506	0.881 (0.605–1.281)
LDL		–0.058	0.060	0.931	0.335	0.944 (0.840–1.061)
CRP		<0.001	<0.001	0.287	0.592	1.000 (1.000–1.000)
Gensini score		0.014	0.001	91.991	<0.001***	1.014 (1.011–1.017)
High Gensini score		0.775	0.113	47.381	<0.001***	2.171 (1.741–2.707)

High Gensini score: >74.8605; SGLT2, sodium-dependent glucose transporters 2; WBC, white blood cell; HbA1c, glycosylated hemoglobin A1c; NT-proBNP, N-terminal pro-brain natriuretic peptide; HDL, high-density lipoprotein; LDL, low-density lipoprotein; CRP, C reactive protein; B, regression coefficient; SE, standard error; Wald χ^2^, Wald Chi-square test statistic; HR, EXP(B); 95% CI, 95% confidence interval; *p < 0.05, **p < 0.01, ***p < 0.001.

**Table 5.** **Multivariate Cox analysis results**.

Variable	Β	SE	Wald χ^2^	*p*	HR (95% CI)
Gensini score	0.013	0.001	80.871	<0.001***	1.013 (1.010–1.016)
NT-proBNP	<0.001	<0.001	9.428	0.002**	1.000 (1.000–1.000)
Lymphocyte	0.012	0.005	6.350	0.012*	1.012 (1.003–1.022)
SGLT2	0.484	0.214	5.111	0.024*	1.623 (1.067–2.469)
Statins	–0.451	0.175	6.641	0.010*	0.637 (0.452–0.898)

NT-proBNP, N-terminal pro-brain natriuretic peptide; SGLT2, sodium-dependent glucose transporters 2; B, regression coefficient; SE, standard error; Wald χ^2^, Wald Chi-square test statistic; HR, EXP(B); 95% CI, 95% confidence interval; *p < 0.05, **p < 0.01, ***p < 0.001.

**Table 6.** **Multivariate Cox analysis results with high Gensini score**.

Variable	Β	SE	Wald χ^2^	*p*	HR (95% CI)
High Gensini score	0.820	0.126	42.289	<0.001***	2.270 (1.773–2.907)
NT-proBNP	<0.001	<0.001	5.276	0.022*	1.000 (1.000–1.000)
Lymphocyte	0.011	0.005	5.453	0.020*	1.011 (1.002–1.021)
SGLT2	0.504	0.214	5.533	0.019*	1.655 (1.088–2.518)
Statins	–0.654	0.173	14.304	<0.001***	0.520 (0.370–0.730)

High Gensini score: Gensini score >74.8605; NT-proBNP, N-terminal pro-brain natriuretic peptide; SGLT2, sodium-dependent glucose transporters 2; B, regression coefficient; SE, standard error; Wald χ^2^, Wald Chi-square test statistic; HR, EXP(B); 95% CI, 95% confidence interval; *p < 0.05, ***p < 0.001.

### 3.4 Correlation between Gensini Score and Major Adverse Cardiac and Cerebrovascular Events

**Fig. 2.**
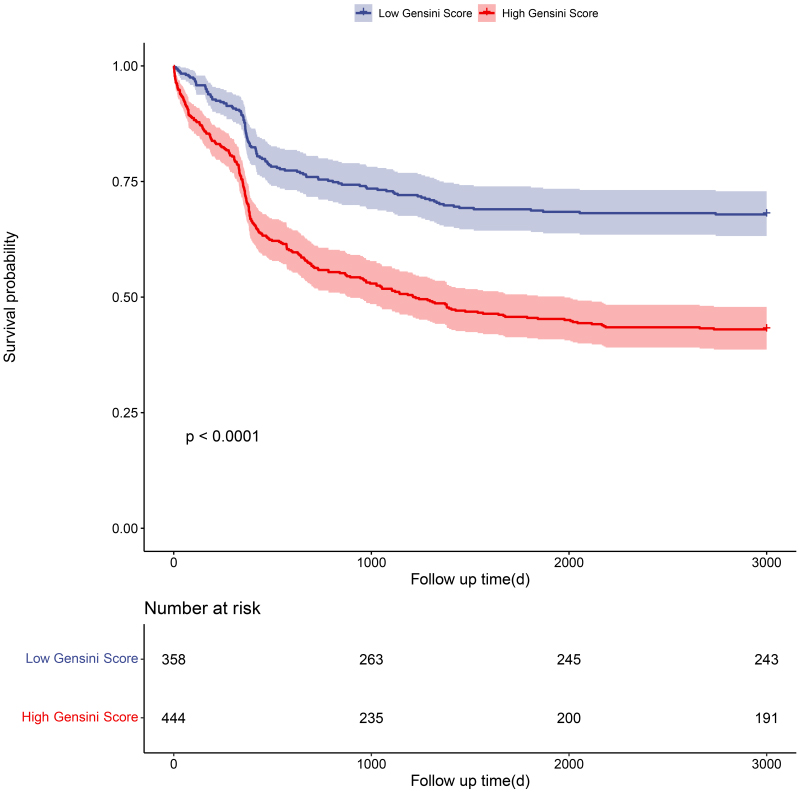
**Kaplan-Meier curves of patient groups with low or high Gensini scores**.

The patient cohort was followed for 3000 days (382.5–3000). Patients with high Gensini score had a significantly increased risk of MACCEs (HR 2.171, 95% CI: 1.741–2.707, *p* < 0.001). Kaplan-Meier analysis was performed on the low and high Gensini score groups (Fig. 2). This revealed a higher incidence of MACCEs in the high Gensini score group than in the low Gensini score group (log rank *p* < 0.001).

## 4. Discussion

A total of 802 DM patients with CHD were enrolled in this retrospective study aimed at investigating the risk factors and incidence of MACCEs using ROC curves, multivariate Cox regression analysis and Kaplan-Meier analysis. The results suggest the Gensini score is a predictive factor for MACCEs in patients with DM and CHD, with a high score being predictive of an increased incidence of MACCEs. Complications from DM have received increased clinical attention in recent years [14, 15, 16]. In the present study, long-term follow-up and in-depth analysis of the risk variables for MACCEs were conducted in patients with both DM and CHD, thus providing a new perspective and basis for the treatment and prognostication of clinical DM complications.

Atherosclerosis can lead to vascular-lumen stenosis and even to insufficiency of the local blood supply and tissue necrosis. It is the main pathological basis of CHD, which is closely linked to its occurrence and severity [17]. The increasing prevalence of DM will lead to a higher incidence of MACCEs in the coming years. One study examined data from 57 articles involving 4,549,481 Type 2 DM patients. The results from 53 of these studies (4,289,140 Type 2 DM patients) suggested that 32.2% of patients had cardiovascular disease, including 29.1% with atherosclerosis and 21.2% with CHD [18]. Furthermore, a meta-analysis found that Type 1 DM patients are also at increased risk of MACCEs [19]. Previous research has shown that DM is a risk factor that can induce the development of atherosclerosis and accelerate its progression [11, 20, 21]. A recent review also showed that diabetic patients experience arteriosclerotic progression earlier and to a greater extent than non-diabetics [22]. Gender, obesity, advanced age, high blood pressure, high fat and high sugar intake are all risk factors for both DM and atherosclerosis. The cellular effects of these risk factors are associated with endothelial dysfunction and increased inflammation [20]. Alteration of lipid metabolism is a risk factor and characteristic feature of atherosclerosis [11]. In the present study, however, patients with high Gensini score had higher levels of HbA1c, while patients with a low Gensini score had a higher mean age. Moreover, there were no significant differences between high and low Gensini score patients in terms of gender, hypertension and LDL level. Cox regression models were used to evaluate the effects of gender, age, HbA1c, hypertension and LDL. The results of this analysis showed they were not significant confounding factors.

The Gensini score is used in clinical practice to assess the severity of CAD. A number of trials have demonstrated the Gensini score is correlated with a variety of coronary artery risk factors. Furthermore, it can predict the risk factors and the severity of complex coronary artery lesions, thus playing an important role in the prediction of clinical prognosis [23]. The Gensini score can also predict for an increased incidence of MACCEs. Therefore, in order to reduce the occurrence of MACCEs in diabetes it is critical to obtain the Gensini score on the blood vessels of DM patients as early as possible. The Gensini score has attracted more and more attention in recent years. A clinical trial found a significant positive correlation between the direct bilirubin level and Gensini score [24]. A study involving 435 patients undergoing coronary angiography found the mean platelet volume was significantly and positively correlated with the Gensini score [25]. Another clinical study found the epicardial adipose tissue thickness of the left anterior descending artery (EATLAD) was greater in CAD patients compared to non-CAD individuals. Furthermore, the EATLAD was closely correlated with CAD and the Gensini score [26]. Yet another study showed that aortic *NLRP3* gene expression was significantly related to the Gensini score [27]. The Gensini score also has important prognostic value in cardiovascular and cerebrovascular diseases, nephropathy, as well as several other diseases. A study of 536 patients with acute coronary syndrome (ACS) showed significant differences in long-term mortality between patient groups with different Gensini scores [28]. The Gensini score was also an effective prognostic index for assessing long-term mortality. However, there have so far been few studies on the Gensini score in patients with both DM and CHD, and further research on this patient group is required.

In contrast to previous studies, the present work focused on the Gensini score of DM patients with CHD, and had a longer follow-up time than other similar studies. The Gensini score can evaluate the degree of vascular stenosis in patients in a more intuitive and quantitative manner. Furthermore, the Gensini score is able to predict a higher incidence of MACCEs in patients with both DM and CHD, which is important for clinical prognostication. The present study offers a new perspective on the diagnosis, treatment, and prognosis of DM patients with CHD. It does have some limitations, however. First, it was a small retrospective study conducted at a single center. Second, it would be preferable to subdivide into groups more precisely in order to better predict the incidence of MACCEs. Third, because of its design this study could not address the causal relationship between the Gensini score and the occurrence of MACCEs in DM patients with CHD. In the future, multi-center prospectively designed studies with larger sample sizes are needed to further clarify the role of the Gensini score in patients with both DM and CHD. This should lead to better stratification of risk management and to the formulation of corresponding diagnoses and treatment strategies that minimize the incidence of MACCEs and improve prognosis.

## 5. Conclusions

High Gensini score is a risk factor for patients with both DM and CHD, with a high score correlating with a high incidence of MACCEs.

## Data Availability

The data will not be shared because the identified participant information is included in the data.
